# Genome Sequence of *Bacillus subtilis* Strain HUK15, Isolated from Hexachlorocyclohexane-Contaminated Soil

**DOI:** 10.1128/genomeA.00273-16

**Published:** 2016-04-14

**Authors:** Cyrielle Gasc, Jean-Yves Richard, Pierre Peyret

**Affiliations:** a EA 4678 CIDAM, Université d’Auvergne, Clermont-Ferrand, France; b SITA Remediation, Suez, Meyzieu, France

## Abstract

*Bacillus subtilis* strain HUK15 has been isolated from hexachlorocyclohexane (HCH)-long-term-contaminated soil. The genome of strain HUK15 was sequenced to investigate its adaptation toward HCH and its potential capability to degrade the pesticide. Here, we report the annotated draft genome sequence (~4.3 Mbp) of this strain.

## GENOME ANNOUNCEMENT

For a long time, hexachlorocyclohexane (HCH) has widely been used as an insecticide to control agricultural pests around the world (1). Many countries, however, have now restricted or banned its use because of its toxicity and persistence in the environment, but many agricultural and industrial sites remain contaminated (2). Bioremediation has become a relevant and promising approach to cleaning up these polluted sites and thus requires a thorough study of indigenous microbial communities (3). In this context, an aerobic HCH-supplemented liquid cultivation of microorganisms from soil contaminated with HCH in an ancient chemical factory (Huningue, France) has been carried out, and subsequent repeated seeding of individual colonies has led to the isolation of the *Bacillus subtilis* strain HUK15 (SITA Remediation, Suez, France). Further study of this strain has demonstrated its capability to grow in minimal salt medium containing HCH as the sole carbon source, suggesting the degradation of HCH by the strain for its metabolism.

Thus, to gain more insight into strain HUK15 adaptive mechanisms toward HCH and its potential capability to degrade it, the genomic DNA of the strain was sequenced by use of an Illumina HiSeq 2500 platform (CASAVA version 1.8.2). The shotgun sequencing generated 2,723,408 high-quality paired-end reads. All reads were quality trimmed using Trimmomatic (version 0.32) (4) and were then assembled *de novo* with Velvet (version 1.2.10) (5). A total of 92 contigs with an *N*_50_ length of 206 kbp and average length of 46.24 kbp were assembled, the largest contig being 632.69 kbp in length. Total sequence spanning scaffolds represents 4,254,363 bp and has a G+C content of 43.47%. Gene prediction and annotation were performed using the NCBI Prokaryotic Genome Annotation Pipeline (PGAP) (http://www.ncbi.nlm.nih.gov/genome/annotation_prok/), which identified 4,353 coding sequences (CDSs) and 150 pseudogenes.

Currently characterized HCH-degrading bacterial species have been found to degrade HCH via different known pathways, which require the *linA* to *linJ* genes (6). Consequently, a BLAST search within the strain HUK15 genome using the corresponding gene sequences of *Sphingobium indicum* B90A (accession no. AJXQ00000000) was performed. None of these genes or their homologues were found, and no other gene was likely to allow for HCH degradation. Thus, these results, in accordance with cultural observations, might indicate that *B. subtilis* strain HUK15 degrades HCH using other uncharacterized metabolic pathways, as suggested by other studies (7, 8).

### Nucleotide sequence accession numbers.

This whole-genome shotgun project has been deposited in DDBJ/ENA/GenBank under the accession no. LSMU00000000. The version described in this paper is the first version, LSMU00000000.1.
